# Effect of a computer-aided diagnosis system on radiologists' performance in grading gliomas with MRI

**DOI:** 10.1371/journal.pone.0171342

**Published:** 2017-02-03

**Authors:** Kevin Li-Chun Hsieh, Ruei-Je Tsai, Yu-Chuan Teng, Chung-Ming Lo

**Affiliations:** 1 Department of Medical Imaging, Taipei Medical University Hospital, Taipei, Taiwan; 2 Research Center of Translational Imaging, College of Medicine, Taipei Medical University, Taipei, Taiwan; 3 Graduate Institute of Biomedical Informatics, College of Medical Science and Technology, Taipei Medical University, Taipei, Taiwan; Worcester Polytechnic Institute, UNITED STATES

## Abstract

The effects of a computer-aided diagnosis (CAD) system based on quantitative intensity features with magnetic resonance (MR) imaging (MRI) were evaluated by examining radiologists' performance in grading gliomas. The acquired MRI database included 71 lower-grade gliomas and 34 glioblastomas. Quantitative image features were extracted from the tumor area and combined in a CAD system to generate a prediction model. The effect of the CAD system was evaluated in a two-stage procedure. First, a radiologist performed a conventional reading. A sequential second reading was determined with a malignancy estimation by the CAD system. Each MR image was regularly read by one radiologist out of a group of three radiologists. The CAD system achieved an accuracy of 87% (91/105), a sensitivity of 79% (27/34), a specificity of 90% (64/71), and an area under the receiver operating characteristic curve (Az) of 0.89. In the evaluation, the radiologists’ Az values significantly improved from 0.81, 0.87, and 0.84 to 0.90, 0.90, and 0.88 with *p* = 0.0011, 0.0076, and 0.0167, respectively. Based on the MR image features, the proposed CAD system not only performed well in distinguishing glioblastomas from lower-grade gliomas but also provided suggestions about glioma grading to reinforce radiologists’ confidence rating.

## Introduction

Diffuse gliomas are the most frequent primary brain tumors formed of neoplastic cells that display glial cell differentiation. They can be subdivided by the degree of malignancy into grades 2 (low grade) to 4 (high malignancy) on the basis of histopathological and clinical criteria established by the World Health Organization (WHO) [1,2]. Glioblastomas (GBMs), WHO grade 4 tumors, are the most aggressive tumor type with a very poor prognosis [3]. On the contrary, lower-grade gliomas (LGGs, grades 2 and 3) have more-favorable outcomes with mean survival times ranging 2~8 years [4]. Therapeutic approaches for these two groups of tumor also differ. More-aggressive and combination managements including surgery, radiation therapy, chemotherapy, and targeted therapy are always reserved for GBMs [5]. Determining the tumor grade depends on several pathological signatures including cytological atypia, angiogenesis, mitotic activity, and necrosis. However, interpretation of some criteria can vary because their definitions are not precise [6,7], thus resulting in ambiguity and misgrading in up to 30% of gliomas [7–10].

With the assistance of non-invasive diagnostic imaging, the accuracy of estimating the grading of diffuse gliomas has increased by applying magnetic resonance (MR) imaging (MRI) [11,12]. In addition to conventional sequences which offer meaningful tissue contrasts of the entire tumor [13], advances in physiological MR techniques, including diffusion-weighted imaging (DWI), perfusion-weighted imaging (PWI), and MR spectroscopy (MRS), also facilitate more-accurate differentiation of LGGs from GBMs [14–17]. Therefore, to avoid unnecessary misdiagnoses, the role of MRI in the diagnostic imaging of brain tumors cannot be overemphasized.

Due to the success of diagnostic imaging, the workload of radiologists has dramatically increased. Computer-aided diagnosis (CAD) may become a new way to handle the data explosion. The computing power of CAD systems can calculate many quantitative features to describe tumor characteristics in real time and combine them together in an artificial intelligence classifier to provide estimates of tumor types and grades [18–20]. With a quantitative approach, diagnostic procedures can be speeded up and diagnostic errors reduced. Consistent estimations can also provide reliable suggestions to radiologists to avoid invasive procedures for which risks outweigh benefits. To ensure that CAD systems can be used in clinical examinations, previous observer performance studies indicated that determining cancer aggressiveness in prostate MR images was significantly improved with the use of CAD systems [21]. Radiologists' confidence rating in diagnosing malignant breast lesions was reinforced with a CAD system using dynamic contrast-enhanced MRI [22].

This study proposed quantifying image features from histograms and textures of MR images to extract characteristics of LGGs and GBMs. Feature selection and combination with machine learning schemes were then used to establish the CAD approach. With this approach, radiologists' performance in grading gliomas on MR images with and without CAD was explored.

## Materials and methods

### Patient information

#### The Cancer Genome Atlas (TCGA) and the Cancer Imaging Archive (TCIA)

MRI datasets of 34 GBM and 71 LGG patients were obtained from TCIA (http://cancerimagingarchive.net/) of the National Cancer Institute, a portal containing images of TCGA patients for image analysis. Original materials and data provided by TCGA project were collected in compliance with all applicable laws, regulations, and policies for the protection of human subjects. All necessary approvals, authorizations, human subject assurances, informed consent documents, and institutional review board (IRB) approvals were obtained [23]. Images used in this study were generated from three institutes: Thomas Jefferson University, Henry Ford Hospital, and Case Western Hospital.

**Fig 1:**
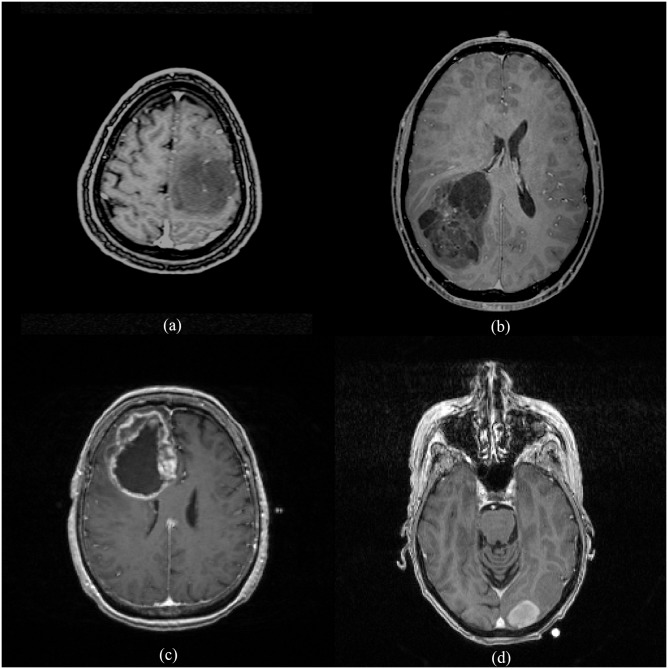
Two cases of low-grade gliomas (a, b) and two cases of glioblastomas (c, d) used in the experiment showing the heterogeneity among these brain tumors. (http://cancerimagingarchive.net/ - "License", the CC BY license (https://creativecommons.org/licenses/by/3.0/).

There were totally 34 GBMs (grade 4) and 71 LGGs (grades 2 and 3) included in this study. In the LGG group, there were 32 oligodendrogliomas, 16 oligoastrocytomas, and 23 astrocytomas. Eighteen oligodendrogliomas were classified into grade 2, and 14 cases were classified into grade 3. Seven cases of oligoastrocytoma were classified into grade 2, and nine cases were classified into grade 3. Among astrocytomas, three cases were classified into grade 2, and 20 cases were classified into grade 3. Therefore, we had totals of 28 grade 2 and 43 grade 3 gliomas in the LGG group (Fig 1).

#### Image analysis and observers’ grading

Imaging features were quantitatively analyzed by the procedures described below. A board-certified neuroradiologist (K.H., with 12 years of experience), who was blinded to the pathological diagnosis, selected the most representative 2D image of each tumor in the contrast-enhanced axial T1-weighted image (T1WI) sequence. Regions-of-interest (ROIs) were then manually drawn to encircle the entire tumor in the selected image using OsiriX. Pixels in the ROI were used for feature analysis and CAD classification.

Determining the effect of the CAD system on radiologists' performance in grading gliomas was the aim of this study. The two-stage procedure design of observers’ grading was very similar to the way CAD systems are used in clinical practice. First, a radiologist performed a conventional reading without the CAD system. A sequential second-read which included the malignancy estimation of the CAD (a continuous likelihood score of 0~1) was provided immediately after conducting the conventional interpretation. Considering the tradeoff between sensitivity and specificity, the radiologist used a 10-point scale of grading to support the endpoints of the receiver operating characteristic (ROC) curve. Tumors with a grading of ≥5 were regarded as GBMs. Each MR image was regularly read by one radiologist out of a group of three radiologists. Three radiologists, including one resident (R, with 2 years' experience in general radiology), one general radiologist (GR, with 11 years' experience in general radiology), and one neuroradiologist (NR, with 12 years in neuroradiology), were involved in this observer study. The experience levels of the radiologists ranged from limited to very experienced.

### CAD approach

#### Global intensity features

**Fig 2:**
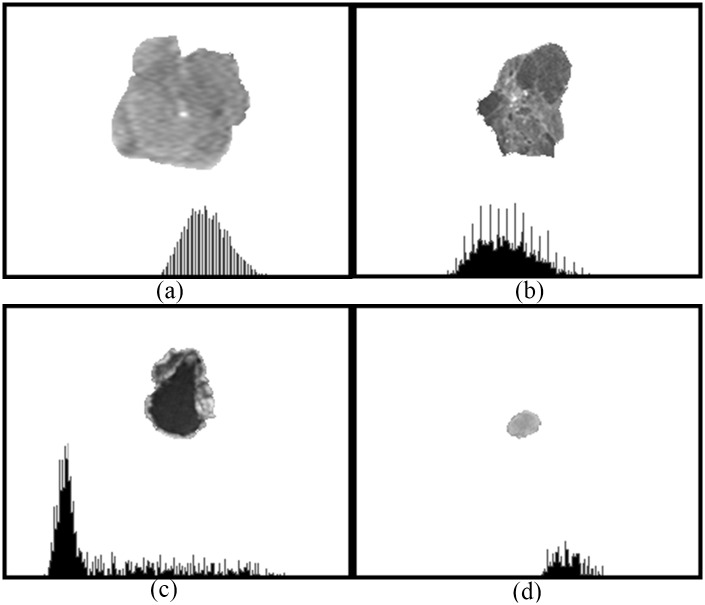
Image histograms showing gray-scale distributions of the delineated tumor areas in Fig 1. (http://cancerimagingarchive.net/ - "License" and the CC BY license (https://creativecommons.org/licenses/by/3.0/, tumor areas in this figure were extracted from original images.).

The gray-scale distribution of pixels in the tumor area reflects different tissue properties expressed by brightness levels such as enhancement in contrast-enhanced MR images. The distribution which is regarded as a function of the brightness probability can be shown as a histogram indicating the global intensity of a tumor (Fig 2).

The histogram moment which describes the shape of a histogram is thus used for characterization in the image analysis [24,25]. In the experiment, differences in the histogram moment between LGGs and GBMs were analyzed as quantitative image features in the CAD system. Four central moments, including the mean, variance, skewness, and kurtosis, were calculated using the following formulas:

$$\text{Mean}=\frac{1}{N}\sum_{i=1}^{N}P_{i},$$ (1)

$$\text{Variance}=\frac{1}{N}\sum_{i=1}^{N}{\left(P_{i}-Mean\right)}^{2},$$ (2)

$$\text{Skewness}=\frac{1}{N}\sum_{i=1}^{N}{\left(\frac{P_{i}-Mean}{Std}\right)}^{3},$$ (3)

and

$$\text{Kurtosis}=\frac{1}{N}\sum_{i=1}^{N}{\left(\frac{P_{i}-Mean}{Std}\right)}^{4};$$ (4)

where *P*_*i*_ is the gray-scale value of a pixel. The mean is the center of a histogram by adding all regional pixels together and dividing the sum by the number of pixels. The extent to which the pixel values are spread out is expressed by the variance, while the skewness estimates the level of symmetry of the value distribution to determine if there is any bias. The estimation of the difference between the histogram center and the two sides is established by comparison to a normal distribution formulated as the kurtosis.

#### Local intensity features

In addition to the global intensity, the local intensity, which describes correlations between adjacent pixel values, was also quantified for tumor characterization. Subtle variations among adjacent pixels are image textures which are used to recognize specific patterns. For MR images, a gray-level co-occurrence matrix (GLCM) [26] was used in the experiment to interpret intensity correlations among pixels with gray-level values. To reduce the computational complexity, the original MR image was quantified into an image, *G*, with reduced intensity bins. Corresponding co-occurrence matrices, *P = [p(i*,*j|d*,*θ)]*, were then generated to present the co-occurrence frequencies of one pixel (*i*) and its neighboring pixel (*j*) at distance *d* and direction *θ*. The parameters: *d* = 1 and *θ* = 0°, 45°, 90°, and 135° were used in the following formulas to extract GLCM texture features.

Autocorrelation=∑i∑j(px−μx)(py−μy)σxσy (5)

$$Contrast=\sum_{n}n^{2}\left\{\sum_{i}\sum_{j}p\left(i,j\right)\right\},\left|i-j\right|=n$$ (6)

$$Correlation=\frac{\sum_{i}\sum_{j}\left(i-\mu_{x}\right)\left(j-\mu_{y}\right)p\left(i,j\right)}{\sigma_{x}\sigma_{y}}$$ (7)

$$Cluster\;prominence=\sum_{i}\sum_{j}{\left(i+j-\mu_{x}-\mu_{y}\right)}^{4}p\left(i,j\right)$$ (8)

$$Cluster\;shade=\sum_{i}\sum_{j}{\left(i+j-\mu_{x}-\mu_{y}\right)}^{3}p\left(i,j\right)$$ (9)

$$Dissimilarity=\sum_{i}\sum_{j}p\left(i,j\right)\left|i-j\right|$$ (10)

$$Energy=\sum_{i}\sum_{j}p{\left(i,j\right)}^{2}$$ (11)

$$Entropy=-\sum_{i}\sum_{j}p\left(i,j\right)\text{log}(p\left(i,j\right))$$ (12)

$$Homogeneity=-\sum_{i}\sum_{j}\frac{1}{1+i-j}p\left(i,j\right)$$ (13)

$$Difference\;variance=\sum_{i}i^{2}p_{x-y}(i)$$ (14)

$$\begin{array}{c} Difference\;entropy=-\sum_{i}^{}p_{x+y}\left(i\right)\text{log}\left(p_{x+y}\left(i\right)\right),\\ \frac{HXY-HXY1}{max\left\{HX,HY\right\}}\;HXY=\left(8\right), \end{array}$$ (15)

$$\begin{array}{cc} Information\;measure\;of\;correlation= & HXY1=-\sum_{i}^{}\sum_{j}p\left(i,j\right)\text{log}\left(p_{x}\left(i\right)p_{y}\left(j\right)\right),\\ & HX=entropy\;of\;p_{x},\;HY=entropy\;of\;p_{y} \end{array}$$ (16)

$$Inverse\;difference\;normalized=\sum_{i}\sum_{j}\frac{1}{1+\left|i-j\right|}p\left(i,j\right)$$ (17)

$$Inverse\;difference\;moment=\sum_{i}\sum_{j}\frac{1}{1+{\left(i-j\right)}^{2}}p\left(i,j\right)$$ (18)

In these equations, *μ*_*x*_, *μ*_*y*_, *σ*_*x*_, and *σ*_*y*_ are the mean and standard deviation (SD) of the marginal distributions of *p*(*i*,*j*|*d*,*θ*).

$$\mu_{x}=\sum_{i}i\sum_{j}p\left(i,j\right),\;\mu_{y}=\sum_{j}j\sum_{i}p(i,j)$$ (19)

$$\sigma_{x}^{2}=\sum_{i}{\left(i-u_{x}\right)}^{2}\sum_{j}p\left(i,j\right),\sigma_{y}^{2}=\sum_{j}{\left(j-u_{y}\right)}^{2}\sum_{i}p\left(i,j\right)$$ (20)

#### Classification

Quantified image features including the global and local intensities were combined in the classifier of the binary logistic regression to distinguish between LGG and GBM tumors. Biopsy-proven pathology was acquired as the gold standard in the classifier. Backward elimination evaluated the distinguishing ability of each feature and excluded redundant features. When the smallest error rate was achieved, the corresponding features were selected to be the most relevant. Meanwhile, leave-one-out cross-validation [27] tested the generalizability of the features with the limited case number. One case was separated from the acquired cases to test the trained model from the remaining cases in an iteration. In the classification result, the likelihood of malignancy of each tumor was presented as a probability. The criterion for determining GBMs was the same as that of radiologists, i.e., ≥ 0.5.

#### Statistical analysis

Performances of both the CAD system and radiologists’ diagnoses are shown using five performance indices: accuracy, sensitivity, specificity, positive predictive value (PPV), and negative predictive value (NPV). The tradeoff between the sensitivity and specificity was calculated and illustrated using an ROC curve. The area under the ROC curve, Az, provided an overall malignancy evaluation using ROCKIT software (C. Metz, University of Chicago, Chicago, IL, USA). In the comparison of radiologists’ performances with and without the CAD, Chi-square tests in SPSS software (vers. 16 for Windows; SPSS, Chicago, IL, USA) and Prism (release 6.0, GraphPad Software, La Jolla, CA, USA) were used.

## Results

**Fig 3:**
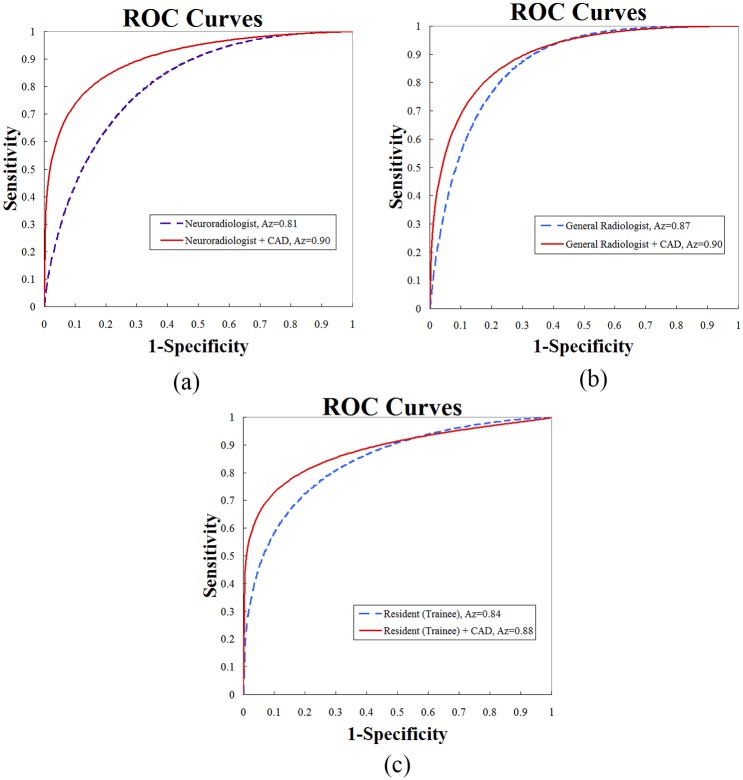
Trade-offs between the sensitivity and specificity of the (a) neuroradiologist, (b) general radiologist, and (c) resident (trainee) with and without the computer-aided diagnosis (CAD) system illustrated by receiver operating characteristic (ROC) curves.

After feature selection, the following image features were combined in the classifier to generate the prediction model: the *Mean*, *Cluster prominence*, *Cluster shade*, *Dissimilarity*, *Energy*, *Entropy*, *Difference variance*, and *Inverse difference normalized*. The most useful imaging feature for the differentiation of GBMs from lower-grade gliomas was *Correlation*, which is a measure of gray level linear dependence between a pixel and its neighbors at specified positions. The *Correlation* achieved an accuracy of 82%. With the complementary power of various image features, the performance of the CAD system achieved an accuracy of 87% (Table 1). *Cluster prominence* and *Cluster shade* are measures of the lack of symmetry in gray-scale distributions, while *Entropy* and *Inverse difference normalized* estimate whether spatial patterns in tissues are heterogeneous and the loss of homogeneous texture. With respect to the first diagnosis of the observers, the three radiologists assessed the MRI appearances with accuracies of 72%, 73%, and 74%. Considering the effect of the assistance from the CAD system, the overall performance indices of three radiologists improved, including the accuracy, sensitivity, specificity, PPV, NPV, and Az. The highest improvement in accuracy was from 72% to 81%, although the improvement was not significant under the predefined threshold of 0.5. However, Az values, representing the overall performance between sensitivity and specificity, significantly improved from 0.81, 0.87, and 0.84 to 0.90, 0.90, and 0.88 with *p* = 0.0011, 0.0076, and 0.0167, respectively. The corresponding ROC curves are illustrated in Fig 3.

**Table 1:** Three radiologists’ performances of the first diagnosis and a subsequent assessment with computer-aided diagnosis (CAD) in the classification of low-grade gliomas (LGGs) and glioblastomas (GBMs).

	Accuracy	Sensitivity	Specificity	PPV	NPV	Az
CAD	87% (91/105)	79% (27/34)	90% (64/71)	79%(27/34)	90%(64/71)	0.89
NR	72% (76/105)	68% (23/34)	75% (53/71)	56% (23/41)	83% (53/64)	0.81
NR + CAD	81% (85/105)	76% (26/34)	83% (59/71)	68% (26/38)	88% (59/67)	0.90
*p* value	0.1420	0.4175	0.2174	0.2595	0.3939	0.0011*
GR	73% (77/105)	88% (30/34)	66% (47/71)	56%(30/54)	92%(47/51)	0.87
GR + CAD	78% (82/105)	88% (30/34)	73% (52/71)	61%(30/49)	93% (52/56)	0.90
*p* value	0.4210	1.0000	0.3611	0.5601	0.8906	0.0076*
R	74%(78/105)	76%(26/34)	73%(52/71)	58%(26/45)	87%(52/60)	0.84
R + CAD	78%(82/105)	82%(28/34)	76%(54/71)	62%(28/45)	90%(54/60)	0.88
*p* value	0.5169	0.5486	0.6996	0.6670	0.5695	0.0167*

* A *p* value of <0.05 indicates a statistically significant difference.

NR, neuroradiologist; GR, general radiologist; R, resident (trainee); PPV, positive predictive value; NPV, negative predictive value; Az, area under the receiver operating characteristic curve.

**Fig 4:**
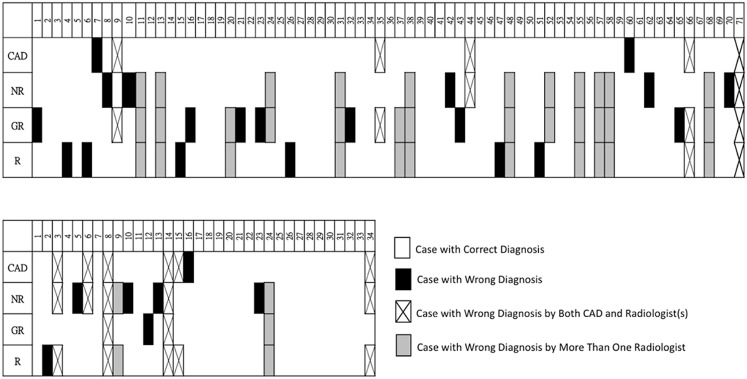
Complete list of misdiagnosed cases in both the low-grade glioma (LGG; upper row) and glioblastoma (GBM; lower row) groups. Misdiagnosed cases are marked in the figures. Many cases were misdiagnosed by more than one radiologist, especially in the LGG group (gray color). (CAD, computer-aided diagnosis; NR, neuroradiologist; GR, general radiologist; R, resident).

**Fig 5:**
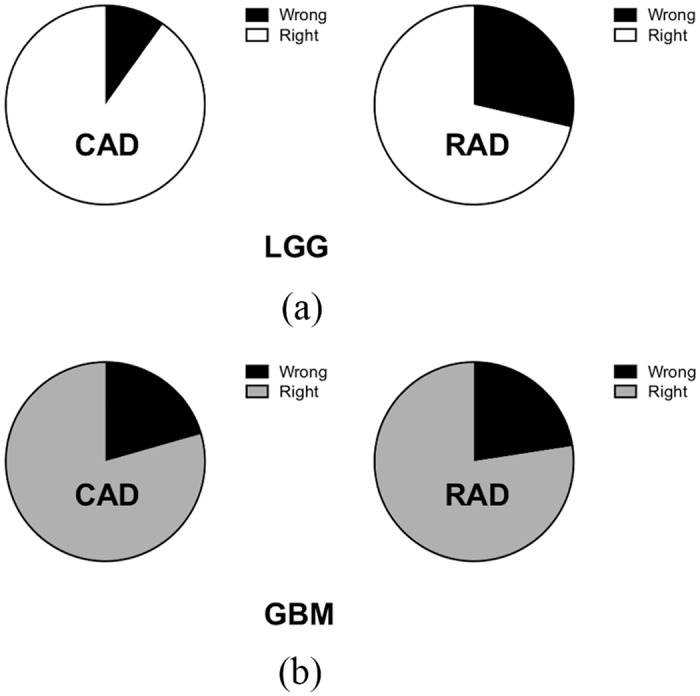
Misdiagnosis percentages of the computer-aided diagnosis (CAD) system and radiologists in both the low-grade glioma (LGG) and glioblastoma (GBM) groups. (a) Remarkably higher misdiagnosis rates of radiologists than that of the CAD system were noted in the LGG group. (b) Similar misdiagnosis rates of both radiologists and CAD were noted in the GBM group. (RAD: radiologist-based diagnosis).

Total numbers of misdiagnosed cases by the three radiologists at the first assessment were 29 (neuroradiologist; NR), 28 (general radiologist; GR), and 27 (resident; R) (Fig 4). Among them, three cases were misdiagnosed by the CAD system and all radiologists. One of them was an LGG and the other two were GBMs. Overlapping numbers of misdiagnosed cases between the CAD system and the three radiologists were seven (NR), six (GR), and seven (R). The CAD system made wrong diagnoses of seven GBMs (20.6%, 7/34) and seven LGGs (9.8%, 7/71). A relatively poorer performance of the CAD system in recognizing GBMs than LGGs (*p* = 0.14, Fig 5) was found. The three radiologists made wrong diagnoses of totally 23 GBMs (22.5%, 23/102) and 61 LGGs (28.6%, 61/213). The radiologists had relatively poorer performances in recognizing LGGs than GBMs (*p* = 0.28, Fig 5). The CAD system had a comparable accuracy to that of the radiologists in recognizing GBMs. However, the radiologists had significantly poorer performances than the CAD system in recognizing LGGs (*p* = 0.001, Fisher’s exact test, Fig 5).

More than half of the cases which were correctly diagnosed by the CAD system but misdiagnosed by radiologists were misdiagnosed by more than one radiologist including 13 LGGs and two GBMs. The common imaging characteristics of these 13 LGG cases were vivid enhancement, obvious necrosis/cystic components, and an irregular or ill-defined tumor margin, and most (10/13, 76.9%) of them were grade 3 gliomas. The other two GBM cases had well-defined tumor margins and less-prominent peritumoral edema.

## Discussion

Brain MRI examinations provide a non-invasive way to interpret tumor characteristics for evaluating the tumor type and grade. Extracting global and local intensity features describing the gray-scale distribution of tissues in the tumor area is useful in interpreting heterogeneous patterns in MR images. This study implemented a CAD system to differentiate LGGs from GBMs based on image intensity features. The performance achieved an accuracy of 87% (91/105), a sensitivity of 79% (27/34), a specificity of 90% (64/71), and an Az of 0.89. The results may be good enough to provide diagnostic suggestions to radiologists. Upon combining the features, the CAD system achieved the highest classification performance. The heterogeneity of a tumor can be highlighted by the CAD system to alert clinicians to aggressive types. The significant image features (*p*<0.05) in distinguishing GBMs from LGGs were listed in a previous study [28]. To evaluate its clinical value, three radiologists with limited experience (2 years as a resident) to very experienced (12 years as a neuroradiologist) were involved in the observer experiment. In the first reading without the CAD system, the three radiologists achieved accuracies of 72%, 73%, and 74% and Az values of 0.81, 0.87, and 0.84, respectively. In the subsequent second reading with malignancy estimations by the CAD system, accuracies respectively improved to 81%, 78%, and 78%. Especially for the Az, improved values of 0.90, 0.90, and 0.88 were significantly better than those of the first reading at *p* = 0.0011, 0.0076, and 0.0167, respectively. These results are in agreement with a previous study which reported that radiologists' confidence ratings were reinforced by a CAD system for diagnosing malignant breast tumors [22]. To the best of our knowledge, this is the first study to explore the effect of CAD on radiologists' performance in grading gliomas. The role of diagnostic imaging of brain tumors is especially crucial to avoid invasive procedures for which risks outweigh benefits.

With respect to the CAD’s performance, the sensitivity of recognizing GBMs was not as good as that of detecting LGGs but was similar to that of the radiologists. Both CAD and radiologists may have been confused by the heterogeneity of GBM cases. For LGG cases, the CAD system achieved a significantly better performance than radiologists (*p* = 0.001). One possible reason is that radiologists possibly preferred assigning a high likelihood of being a GBM to avoid missing a carcinoma. Another reason is that the CAD system truly simultaneously considered more image features and obtained a better malignancy estimation. The quantitative global and local intensity features reflected the difference in the grading of gliomas. Conventional imaging features of grade 2 and 3 gliomas (LGGs) and grade 4 gliomas (GBMs) applied by radiologists are briefly summarized. Grade 2 gliomas usually present as a homogeneous hypointense mass in T1WIs with a circumscribed tumor margin and no enhancement on post-contrast enhanced study. In grade 4 cases, there is always a thick, irregular ring of enhancement surrounding central necrosis. The enhancement may be solid, a ring, nodular, or patchy. Grade 3 gliomas usually present as an infiltrating mass with variable enhancement, ranging from no enhancement to focal or homogeneous enhancement [29]. These features were always applied as diagnosis criteria to differentiate each type of diffuse glioma. However, these imaging characteristics are not quantitative, and can be subjective and biased in some situations when they are applied. Moreover, significant overlap of these imaging characteristics between groups also limits their value as definitive predictors of grade [30,31]. In our study, we found that all radiologists had poorer accuracy in differentiating LGGs and GBMs than did the CAD system, which suggests that these non-quantitative features have important but still limited value. In our results, we also found that radiologists had the poorest performance in recognizing LGGs (Fig 4), especially grade 3 cases. This may be associated with the overlapping imaging presentations of grade 3 gliomas and GBMs. Most radiologists tended to treat cases that presented with vivid enhancement and necrosis as GBMs. These results imply that in cases with equivocal imaging patterns, which always occur with grade 3 gliomas, the CAD system may be helpful in preventing human errors and can reinforce radiologists’ role in make a correct grading of diffuse gliomas. Based on the success, some subgroups presenting different patterns compared to others such as the difference between astrocytomas and oligodendrogliomas may be further classified in a future study. Other brain pathologies including lymphomas, abscesses, tumefactive demyelinating lesions, and metastases can also mimic gliomas or glioblastomas in MRI. Consequently, more prediction models will be investigated for the differential diagnosis of the abovementioned brain pathologies in our future work.

In the present study, only contrast-enhanced T1WIs were used instead of complete MR sequences to estimate the tumor grading. This differs from the clinical practice of most radiologists. The obvious shortcoming of this design is that neither peritumoral edema nor the infiltrating part of the tumor were better depicted in T2-weighted images or fluid-attenuated inversion recovery (FLAIR) sequences than in T1WIs. Using as many image sequences as possible to simulate the clinical practice of radiologists would be ideal. However, with limited image sequences, this study focused on the effect of a CAD system on radiologists' performance for potential clinical application. This limitation may have resulted in some biased interpretation by radiologists, who always check all sequences they have before making a decision. Nevertheless, CE T1WIs still provide much information of tumor characteristics. The key features for differentiating grades 2 and 3 from grade 4 gliomas are necrosis and/or angiogenesis. Necrosis appears as a non-enhanced area within the neoplasm with a signal corresponding to cerebrospinal fluid, which can always be clearly demonstrated in contrast-enhanced T1WIs [12]. Furthermore, the degree of contrast enhancement was found to be linked to the activity of the angiogenesis module within the tumor [32,33]. Since both necrosis and angiogenesis are important criteria used to differentiate GBMs from LGGs, measurements of signal intensities on contrast-enhanced T1WIs should be key determinants distinguishing them. However, further investigation of the role of other MR sequences in the CAD system including T2, FLAIR, PWI, DWI, and MRS is justified.

According to the image features used as conventional diagnostic criteria for differentiating each type of glioma, a limitation of the proposed CAD system is a lack of morphological features describing the circumscribed tumor margin and the infiltration level. Additionally, separating enhanced regions and other regions within the tumor to analyze heterogeneity composed of the irregular ring of enhancement would be useful for further classification. Nevertheless, since the intensity difference used in grading was already implemented in the CAD system and achieved significantly better classification than radiologists, an interactive alarm can be designed in the CAD system that provides evidence it detects rather than just a malignancy probability. Other future work would be a more-powerful CAD system which can classify gliomas into more grades as does the conventional diagnosis. More relevant image features should be added with more brain tumors to have a substantial statistical analysis.

In our study design, the CAD system highlighted some relevant properties of tumors for radiologists with an estimate of the malignancy. However, as the US Food and Drug Administration suggests, CAD is preferred as a second reader after the first read done by a radiologist [34]. The analytical results of CAD can be taken as a good reference before making a final conclusion of an imaging study. With continuous improvements in this system, we believe CAD will work as a good teammate of radiologists. In conclusion, this study evaluated the effect of a CAD system on radiologists' performance in grading gliomas. As a result, the CAD system composed of global and local intensity features assisted the three radiologists in achieving significantly better Az values (*p* = 0.0011, 0.0076, and 0.0167). In particular, image differences between grade 3 gliomas and GBMs were better recognized by the CAD system than by radiologists. In clinical examinations, the proposed CAD system can provide suggestions about glioma grading to reinforce radiologists’ confidence ratings.
